# Fibroblast growth factor-5 promotes spermatogonial stem cell proliferation via ERK and AKT activation

**DOI:** 10.1186/s13287-019-1139-7

**Published:** 2019-01-22

**Authors:** Ruhui Tian, Chencheng Yao, Chao Yang, Zijue Zhu, Chong Li, Erlei Zhi, Junlong Wang, Peng Li, Huixing Chen, Qingqing Yuan, Zuping He, Zheng Li

**Affiliations:** 1 Department of Andrology, the Center for Men’s Health, Urologic Medical Center, Shanghai Key Laboratory of Reproductive Medicine, Shanghai General Hospital, Shanghai Jiao Tong University School of Medicine, 100 Haining Road, Shanghai, 200080 China; 2 Shanghai-MOST Key Laboratory of Health and Disease Genomics, Chinese National Human Genome Center at Shanghai, 250 Bibo Road, Shanghai, 201203 China; 3 Shanghai Key Laboratory for Assisted Reproduction and Reproductive Genetics, Center for Reproductive Medicine, Renji Hospital, School of Medicine, Shanghai Jiao Tong University, 845 Lingshan Road, Shanghai, 200135 China; 4 School of Medicine, Hunan Normal University, Changsha, 410013 Hunan China

## Abstract

**Background:**

Sertoli cells are the most important somatic cells contributing to the microenvironment (named niche) for spermatogonial stem cells (SSCs). They produce amounts of crucial growth factors and structure proteins that play essential roles in the complex processes of male SSCs survival, proliferation, and differentiation. It has been suggested that Sertoli cell abnormalities could result in spermatogenesis failure, eventually causing azoospermia in humans. However, to the end, the gene expression characteristics and protein functions of human Sertoli cells remained unknown. In this study, we aimed to evaluate the effect of fibroblast growth factor-5 (FGF5), a novel growth factor downregulated in Sertoli cells from Sertoli cell-only syndrome (SCOS) patients compared to Sertoli cells from obstructive azoospermia (OA) patients, on SSCs.

**Methods:**

We compared the transcriptome between Sertoli cell from SCOS and OA patients. Then, we evaluated the expression of FGF5, a growth factor which is downregulated in SCOS Sertoli cells, in human primary cultured Sertoli cells and testicular tissue. Also, the proliferation effect of FGF5 in mice SSCs was detected using EDU assay and CCK-8 assay. To investigate the mechanism of FGF5, Phospho Explorer Array was performed. And the results were verified using Western blot assay.

**Results:**

Using RNA-Seq, we found 308 differentially expressed genes (DEGs) between Sertoli cells from SCOS and OA patients. We noted and verified that the expression of fibroblast growth factor-5 (FGF5) was higher in Sertoli cells of OA patients than that of SCOS patients at both transcriptional and translational levels. Proliferation assays showed that rFGF5 enhanced the proliferation of mouse SSCs line C18-4 in a time- and dose-dependent manner. Moreover, we demonstrated that ERK and AKT were activated and the expression of Cyclin A2 and Cyclin E1 was enhanced by rFGF5.

**Conclusion:**

The distinct RNA profiles between Sertoli cells from SCOS and OA patients were identified using RNA-Seq. Also, FGF5, a growth factor that downregulated in SCOS Sertoli cells, could promote SSCs proliferation via ERK and AKT activation.

## Introduction

Male infertility is a common reproductive disorder which contributes to about 10–15% of infertile couples in the world [1, 2]. Azoospermia, consisted of obstructive azoospermia (OA) and non-obstructive azoospermia (NOA), is the major cause of male infertility [3]. OA is caused by obstruction of the reproductive duct, and the patients with OA are considered to have normal spermatogenesis. In contrast with OA, NOA display germ cell reduction or absence by pathological analysis. Sertoli cell-only syndrome (SCOS) is a type of NOA with the most severe impairment of spermatogenesis, diagnosed by the testicular biopsy displaying that seminiferous tubules are lined with only Sertoli cells, with complete depletion of male germ cells. In clinic, however, the diagnosis and treatment of NOA remain a great challenge [3, 4]. Firstly, azoospermia is usually determined by the pathological diagnosis which is mainly dependent on the fine-needle aspiration biopsy. However, the fine-needle aspiration often provides limited testicular tissues for correct histological diagnosis [5, 6]. In addition, the mechanisms of NOA have not been elucidated by far, so the treatment is often ineffective due to the lack of effective treatment target [4, 7].

Spermatogenesis is a complex and well-organized process, which referred to the spermatogonial stem cell (SSCs) differentiation through meiosis to produce mature haploid spermatozoa. Spermatogenesis takes place in the seminiferous tubules and is dependent on the appropriate microenvironment or niche of the tubules [3, 4, 8]. Within the seminiferous tubules, differentiating germ cells stay close to Sertoli cells. As the main support cells, Sertoli cells are involved in all stages of spermatogenesis and are believed to be pivotal to spermatogenesis [4, 8, 9]. Proper gene expression patterns form the basis for Sertoli cell functions and male germ cell differentiation. The abnormal transcriptome of Sertoli cells were considered to be associated with dysfunctions of spermatogenesis, which may cause azoospermia in humans [3]. Although spermatogenesis has been deeply studied, a large number of genes involved in this process are yet unknown. A detailed knowledge regarding the molecular regulations at the transcriptional level in the testis is essential to understand the complex interaction under normal and pathological conditions [9, 10].

In this regard, increasing attentions have been paid to explore the genetic and molecular mechanisms of spermatogenesis and male infertility [3, 11, 12]. The development of gene expression profiling techniques, including ESTs and microarrays, enabled us to discover complex gene expression profiles in the testes [13–16]. Recently, RNA sequencing (RNA-Seq) has been proved to be a cost-effective and high-throughput mean to yield and analyze the transcriptome in specific tissues or cells [17, 18]. Laiho et al. analyzed the gene expression differences during the first wave of murine spermatogenesis using the SOLiD4 next-generation sequencing platform. The total of 26,000 genes and 2494 differentially expressed genes (DEGs) was identified in mouse testis at postnatal days 7, 14, 17, 21, and 28, which were specific to different developmental time points and postulated to be involved in testicular development [19]. In another study, a total of 18,837 genes was detected in infant (postnatal day 6), juvenile (postnatal day 28), and adult (postnatal day 70) mice. However, gene expression pattern in infant mouse testes was distinct from that in juvenile and adult. There were 1722 upregulated and 2712 downregulated expressed genes in juvenile mouse testes compared with those of infant mice, while there were 190 upregulated and 185 downregulated expressed genes in adult testes relative to those of juvenile mice. In addition, it was found that the MAPK, Hedgehog, and Wnt signaling pathways were significantly involved in different developmental stages of mouse spermatogenesis [20]. Margolin et al. characterized gene expression from the testes of juvenile mice of different ages and defined more than 1000 meiotically expressed genes. They also developed a deconvolution algorithm to computationally determine cell type-specific gene expression and estimated gene expression levels in somatic cells, pre-meiotic spermatogonia, spermatocytes, and spermatozoa [21]. In human study, the testis and 26 other tissues were analyzed and more than 1000 genes were revealed to be enriched in the testis versus all other tissues [22]. However, a gene expression analysis on total RNA was prepared from the whole testis containing different cell types. Furthermore, the composition of different cells varies among different stages and in various abnormal spermatogenesis testes [19, 20]. Therefore, the whole testis gene expressions cannot distinguish specific mRNA information of certain cell types, which will mislead us to understand the spermatogenesis mechanisms. In addition, as the main supporting cells, human Sertoli cell gene expression profiles have not been established.

In this study, we have for the first time reported that 308 genes were distinctly expressed in human Sertoli cells between SCOS and OA patients. In these DEGs, we found that fibroblast growth factor-5 (*FGF5*) was downregulated in human Sertoli cells of SCOS patients compared to that of OA patients. It has been reported that FGF5 acts as a mitogen to stimulate the proliferation of mesenchymal fibroblasts and mesenchymal stem cells [23]. As for the reproductive system, it has been demonstrated that FGF5 was mainly expressed in mouse Sertoli cells and also displayed plenty of expressions in goat testes [24, 25]. However, the function of FGF5 in male reproduction regulation remains unclear. Proliferation assays and molecular detections demonstrated that rFGF5 promoted the proliferation of mouse SSCs via activating ERK and AKT, subsequently enhancing Cyclin A2 and Cyclin E1. The study could offer novel mechanisms controlling the fate determinations of human Sertoli cells, and it could provide new targets for therapy of azoospermia.

## Materials and methods

### Procurement of testicular biopsies from OA patients with normal spermatogenesis and NOA patients

Testicular biopsies were obtained from 10 OA and 10 SCOS patients who underwent microdissection TESE (MD-TESE) at Shanghai General Hospital. Patients with OA were caused by inflammation and vasoligation but not by congenital absence of the vas deferens (CBAVD) or other diseases including cancer. Patients with NOA were confirmed by histological analysis, and patients with reproductive congenital disease such as Klinefelter syndrome and genomic AZF deletions, or other diseases including cancer were excluded in this study. Information of patients was provided in Table 1.

**Table 1:** Description of the patients in the study

Parameter	OA	SCOS	*P* value
Age (years)	30.2 ± 3.91	28.8 ± 3.88	0.43
Duration of infertility (months)	32.4 ± 12.71	34.4 ± 18.01	0.78
Testis volume (ml)	15.0 ± 3.55	7.2 ± 2.63	< 0.0001
Testosteron (ng/ml)	3.8 ± 1.50	3.9 ± 1.80	0.8755
LH (mIU/ml)	4.8 ± 1.43	11.8 ± 2.19	< 0.0001
FSH (mIU/ml)	5.0 ± 3.03	23.0 ± 6.10	< 0.0001

### Isolation of Sertoli cells from NOA and OA patients

Testicular biopsies obtained from patients were washed three times aseptically in Dulbecco modified Eagle medium (DMEM). Sertoli cells were isolated from human testis biopsies using the two-step enzymatic digestion according to a procedure we have previously described [4]. Firstly, seminiferous tubules were obtained after being treated with enzymatic solution containing collagenase type IV (2 mg/ml) and DNAse I (10 μg/ml) in DMEM/F12 at 37 °C for 10 min. After washing to remove interstitial cells, Sertoli cells were obtained using a second enzymatic digestion with 4 mg/ml collagenase IV, 2.5 mg/ml hyaluronidase (Sigma), 2 mg/ml trypsin (Sigma), and 1 μg/μl DNAse I and followed by differential plating. Briefly, cell suspensions were seeded into culture plates in DMEM/F12 (Gibco) supplemented with 10% FBS and incubated at 34 °C in 5% CO2 for 3 h. After incubation, the media containing male germ cells were removed, and Sertoli cells were attached to culture plates. The viability of freshly isolated Sertoli cells was assessed by exclusion of trypan blue staining, and Sertoli cells were identified by RT-PCR and immunocytochemistry as described below.

### RNA isolation, library construction, and Illumina sequencing

Total RNA was extracted from human Sertoli cells using RNeasy Mini Kit (Qiagen) and treated with DNase I (Qiagen) to remove genomic DNA. RNA concentration and integrity was measured using Agilent 2100 Bioanalyzer system (Agilent technologies). Equal amount of total RNA of Sertoli cells from five patients was pooled together into one sample containing 1 μg RNA for use in RNA-Seq. A total of 20 patients with OA and SCOS were included in RNA-Seq.

The mRNA was enriched using MicroPoly(A)Purist™ (Ambion). Then, the RNA-Seq library was prepared with 5 ng purified mRNA using Illumina® TruSeq® RNA Sample Preparation Kit v2 (Illumina). The mRNA was cleaved into short fragments with Random Primers and fragment Mix, followed by the first strand of cDNA synthesis using First Strand Master Mix. Then, Second Strand Master was added to synthesize the second strand. The library was amplified with 15 cycles of PCR. The final library used for RNA-Seq was purified using AMPure XP Beads.

In order to ensure quality, RNA library preparation was quantified by Qubit, followed by being detected by High-sensitivity DNA chip (Thermo). Then, the cDNA library of 10 ng was used for cluster generation using the TruSeq PE Cluster Kit v3 (Illumina) on a CBot instrument. Thereafter, the library was sequenced on an Illumina HiSeq 2500 instrument with 150-bp paired end (PE) reads.

### Raw data pre-processing

Raw sequenced reads were saved as FASTQ format. Raw reads were filtered out using FASTX-Toolkit (http://hannonlab.cshl.edu/fastx_toolkit/) to obtain high-quality clean reads, on which the subsequent analysis is based. Briefly, adaptor sequences of the raw reads were removed using fastx_clipper. Trimming was also performed using fastq_quality_filter, which included the removal of reads with ambiguous nucleotide, of reads with low-quality nucleotide (*q* < 20), and of reads shorter than 50 bp.

### Mapping and gene expression analysis

The clean reads above were mapped to the reference genome (hg19, http://asia.ensembl.org) using TopHat v2.0.8b software. Then, gene counts were acquired using HTSeq v0.6.1 (https://htseq.readthedocs.io/en/release_0.11.1/). DEGs were analyzed using DESeq package in R (R package, http://www.bioconductor.org/). Gene counts were transformed to pseudoreads, which was the standardized gene expression level. DEGs have three criteria: adjusted *P* value< 0.05, baseMean > 100, and foldchange > 2 or < 1/2. Here, baseMean represents mean expression level (after DESeq normalization) for a gene of all compared samples. Hierarchical cluster analysis was performed with DESeq R package (http://www.r-project.org/).

### Gene ontology annotation and pathway analysis

Gene ontology (GO) annotation analysis was performed on the DEGs in Sertoli cells from OA and SCOS patients using Blast2GO software. The results were categorized with respect to biological process, molecular function, and cellular component at level 2. Pathway analysis was performed by uploading these genes to DAVID (http://david.abcc.ncifcrf.gov/).

### RT-PCR and quantitative real-time PCR

RT-PCR was performed to examine the expression of Sertoli cell-specific genes, including *GATA4*, *WT1*, *FSHR*, and *ABP*, et al. PCR products were separated by electrophoresis on 1.2% agarose gels, and the gels were exposed to chemiluminescence (Chemi-Doc XRS, Bio-Rad, Hercules, CA). Real-time PCR reactions were performed using a Thermal Cycler DiceTM Real-time System (Takara) and the SYBR Premix Ex TaqTM reagents kit (Takara). Real-time PCR was used to verify the expression profiles of DEGs detected by RNA-Seq. The expression level values were normalized to those of ACTB as a control. Relative fold changes of mRNA expression were calculated using the ΔΔCt method, and the values were expressed as 2^−ΔΔCt^.

### Protein extraction and Western blot

Total proteins were extracted from the cultured cells homogenized in RIPA lysis buffer (Santa Cruz). Thirty micrograms of proteins was electrophoresed on the SDS-PAGE and transferred to polyvinylidene difluoride membranes. After blocking with 5% milk, membranes were incubated with antibodies against FGF5 (R&D, MAB2371, dilution 1:2000), p-ERK1/2 (CST, 4370, dilution 1:2000), ERK1/2 (CST, 4695, dilution 1:1000), p-AKT (CST, 4060, dilution 1:2000), AKT (CST, 4691, dilution 1:1000), p-CREB (Santa Cruz, sc-81486, dilution 1:1000), c-fos (Santa Cruz, sc-447, dilution 1:600), STAT3 (Santa Cruz, sc-8019, dilution 1:200), PCNA (Santa Cruz, sc-25280, dilution 1:600), Cyclin A2 (Proteintech, 18202-1-AP, dilution 1:2000), Cyclin E1 (Proteintech, 11554-1-AP, dilution 1:2000), or ACTB (beta-actin) (Santa Cruz, sc-47778, dilution 1:500) at 4 °C overnight. Membranes were incubated with secondary antibodies for 1 h at room temperature. After incubation with the corresponding secondary antibodies, the blots were detected by the enhanced chemiluminescence (Chemi-Doc XRS, Bio-Rad).

### Cell culture

The C18-4 cells were used in the in vitro experiment. The C18-4 cell line was established by mouse SSCs transfection. The C18-4 cells possess phenotypic characteristics similar to SSCs as evidenced by the expressions of various markers for SSCs, including OCT-4, GFRA1, and PLZF [26]. C18-4 cells were cultured with DMEM/F12 medium containing 10% FBS and 2 mM glutamine and were passaged when they reach 80% confluence. The cells were cultured in six-well plates. The cells were treated by rFGF5 (R&D) with varied concentrations, or control culture medium. As for agent preparation, rFGF5 was dissolved and diluted in PBS containing 0.1% BSA, while control group contained equal volume of PBS as the rFGF5-treated group.

### Cell proliferation assays

In proliferation experiment, for CCK8 assay, the C18-4 cells were seeded in the 96-well microplates containing 200 μl culture medium. After the cell attachment, the cells were starved in serum-free medium for 16 h. The medium was changed by culture medium with rFGF5 (R&D) with varied concentrations, or control culture medium. After 1 to 5 days of culture, CCK-8 medium (Dojindo) was added to the cells. The optical density (OD) for each well was measured at 450 nm using a microplate reader (Bio-Rad Model 550).

### EDU incorporation assay

For EdU assay, the C18-4 cells were seeded in 96-well culture plates with DMEM/F-12 containing 1% FBS. The cells were starved in serum-free DMEM/F-12 for 24 h, followed by the treatment with or without 100 ng/ml rFGF5 and cultured for another 48 h. Then, the culture medium was replaced by 100 μl DMEM/F-12 containing 10 μl EdU, and the proliferation potential of cells was detected according to the manufacturer’s instruction.

### Phospho Explorer Array

The C18-4 cells were seeded in six-well culture plates with DMEM/F-12 containing 10% FBS. The cells were starved in serum-free DMEM/F-12 for 16 h, followed by the treatment with 100 ng/ml rFGF5 and cultured for 15 min. Total proteins were extracted from the cultured cells before and after rFGF5 treatment and then treated with lysis buffer (full moon) The Phospho Explorer Array was detected according to the manufacturer’s instruction (Phospho Explorer Antibody Array, full moon).

### Immunocytochemistry

After fixation with 4% paraformaldehyde (PFA) for 30 min, the expression of GATA4, WT1, and FGF5 was detected in primary isolated Sertoli cells using immunocytochemistry. Briefly, the cells were permeabilized with 0.4% Triton/X-100 in PBS. Blocking was performed in 1% bovine serum albumin (BSA) for 1 h prior to incubation with primary antibodies, including GATA4 (Santa Cruz, dilution 1:100), WT1 (Santa Cruz, dilution 1:100), and FGF5 (R&D, MAB2371, dilution 1:600). The cells were incubated with primary antibodies overnight at 4 °C, followed by Alexa Fluor 488-labeled secondary antibody or Alexa Fluor 594-labeled secondary antibody at a 1:200 dilution for 2 h at room temperature. DAPI (4′-6-diamidino-2-phenylindole) was used to label cell nuclei, and the cells were observed using a fluorescence microscope (Leica).

### Histological examination

The testicular biopsies were fixed in Bouin’s solution overnight, embedded in paraffin, and sectioned at 5 μm thickness. The sections were stained with hematoxylin and eosin (H&E) and observed for the tissue structure under a microscope (Leica).

### Statistical analysis

GraphPad Prism 5 was employed for statistical analysis. All the values were presented as mean ± SD, and statistically significant differences (*P* < 0.05) between SCOS and OA patients were determined using analysis of variance (ANOVA) and a two-tailed *t* test.

## Results

### Identification of human Sertoli cells from SCOS and OA patients

**Fig. 1:**
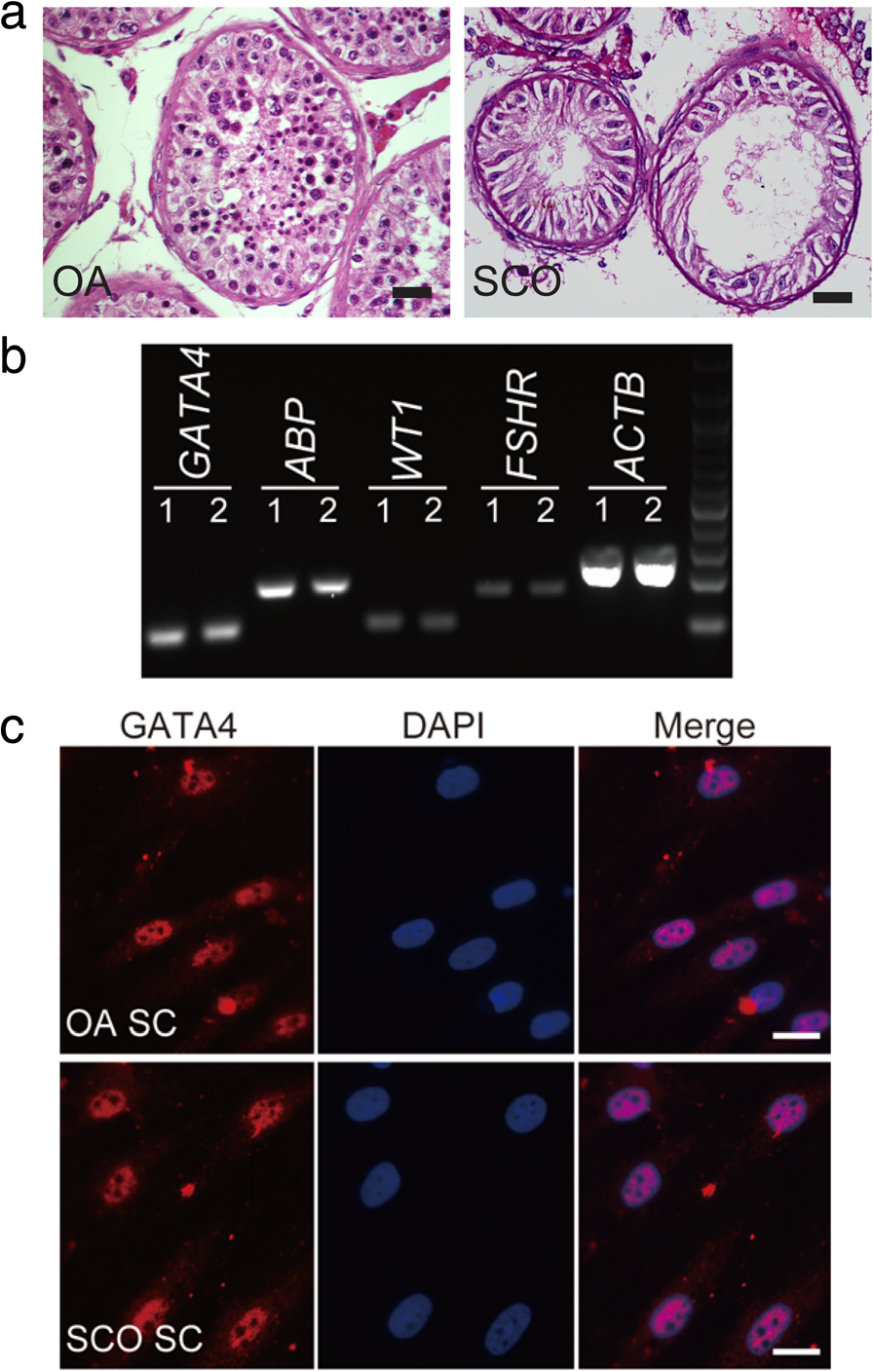
Morphology of the testis from OA and SCOS patients and identifications of isolated human Sertoli cells. **a** H&E staining illustrated the morphology of testicular tissues from OA (left panel) and SCOS patients (right panel) (scale bar = 20 μm). **b** RT-PCR showed that the human Sertoli cells isolated from OA (1) and SCOS (2) patients’ testes expressed transcripts of *GATA4*, *ABP*, *WT1*, and *FSHR*. *ACTB* (actin beta) was used as a loading control of total RNA. **c** Immunohistochemistry reveals that GATA4 is expressed in human Sertoli cells isolated from OA and SCOS patients’ testes (scale bar = 10 μm). Note: OA obstructive azoospermia, SCOS Sertoli cell-only syndrome, SC Sertoli cell

Histological examination showed that Sertoli cells were located on the basement membrane to form seminiferous tubules in the testis from OA patients. There were male germ cells ranging from spermatogonia to spermatids within the seminiferous epithelium. However, the seminiferous epithelium integrity of the testes from OA patients did not completely correspond to the normal testis. As for the testis from SCOS patients, it was found that all germ cells were lost except for a single layer of Sertoli cells at the basement membrane within the tubules (Fig. 1a).

Human Sertoli cells from patients with OA and SCOS were isolated by a two-step enzymatic digestion and followed by differential plating. Specific biomarkers of Sertoli cells were used for identifications of the isolated cells. RT-PCR showed that the isolated cells from both OA and SCOS patients expressed human Sertoli cell-specific genes, including *GATA4*, *ABP*, *FSHR*, and *WT1* (Fig. 1b). Immunocytochemistry revealed that the proposed Sertoli cells were positive for GATA4 (Fig. 1c). The purity of isolated Sertoli cells was assessed to be nearly 100% according to their expression of GATA4.

### Transcriptome of human Sertoli cells

The integrity of total RNA used for RNA-Seq was assessed by denaturing agarose gel electrophoresis. RNA-Seq was performed to generate gene expression profiles of human Sertoli cells from OA and SCOS patients. The summary of RNA-Seq results is displayed in Table 2.

**Table 2:** Summary of RNA-Seq of human Sertoli cells from OA and SCOS patients

Reads	OA-SC-P1	OA-SC-P2	SCOS-SC-P1	SCOS-SC-P2
Total reads	40, 382, 398	42, 555, 605	31, 465, 249	29, 300, 186
Clean data	37,923,822	38,721,246	30,111,972	28,108,329
Percent (%)	93.9	91	95.7	95.9
The comparison between libraries				
Detected genes*(%)	13,759 (55.3)	13,978 (56.2)	13,482 (54.2)	13,449 (54.1)
Total detected genes in SC	14, 618			
Common genes within group	13, 429		13, 050	

*Detected genes means the gene expression levels more than 0.5 cpm (counts per million). And P means pools of samples from patients

**Fig. 2:**
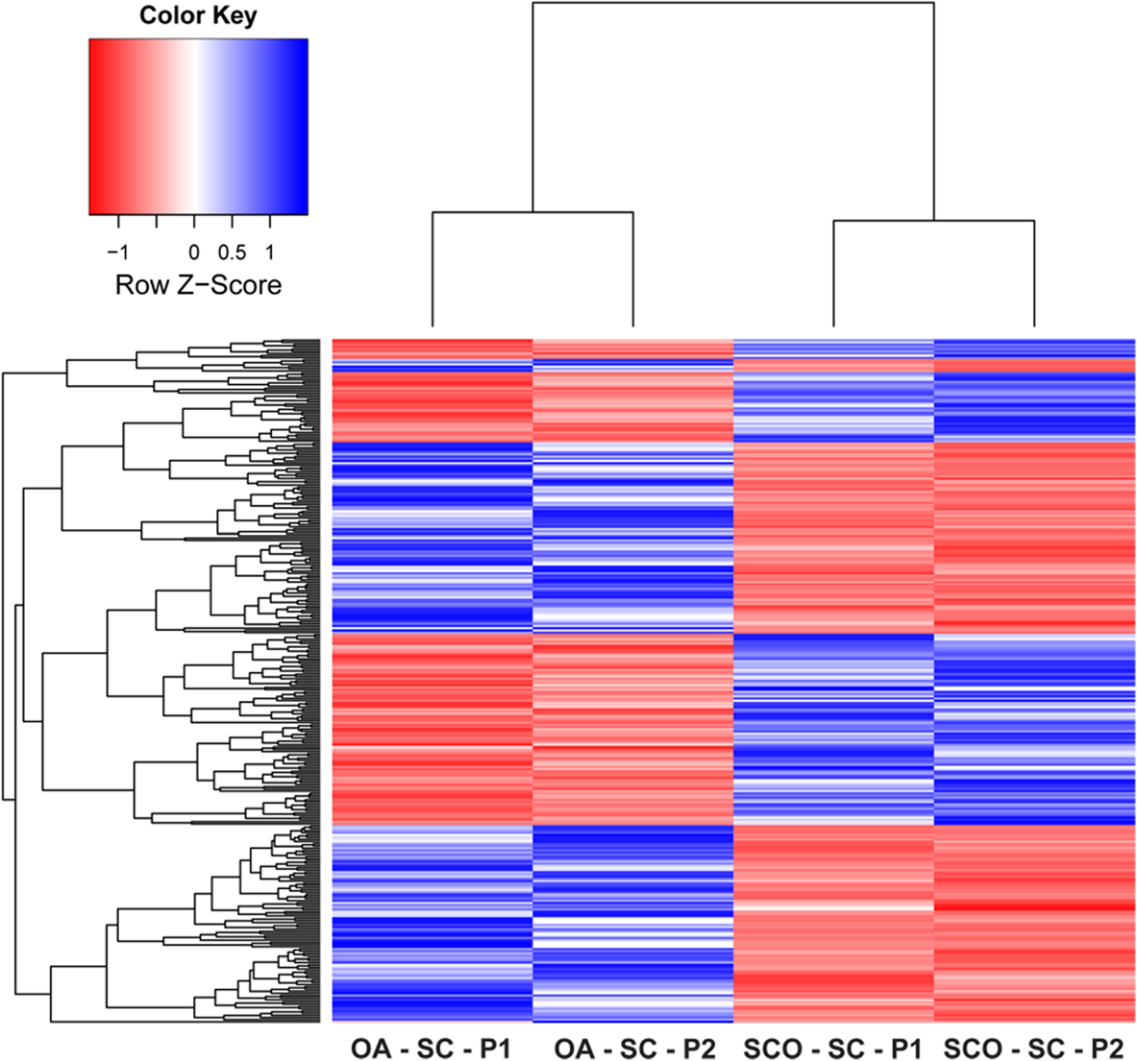
Hierarchical clustering analysis of DEGs. Heatmap consisted of differentially expressed genes of Sertoli cells from OA and SCOS patients. Note: OA obstructive azoospermia, SCOS Sertoli cell-only syndrome, SC Sertoli cell, P pools of samples from patients

Hierarchical cluster analysis was performed to reveal the expression patterns of DEGs between OA and SCOS Sertoli cells. Expression profiling analysis revealed the significant differential expression of 308 genes in Sertoli cells from SCOS patients compared with those from OA patients. Two clusters of the DEGs were evidenced, consisting of 182 down- and 126 upregulated genes in SCOS testes (Fig. 2).

### Quantitative real-time PCR verification of RNA-Seq results

**Fig. 3:**
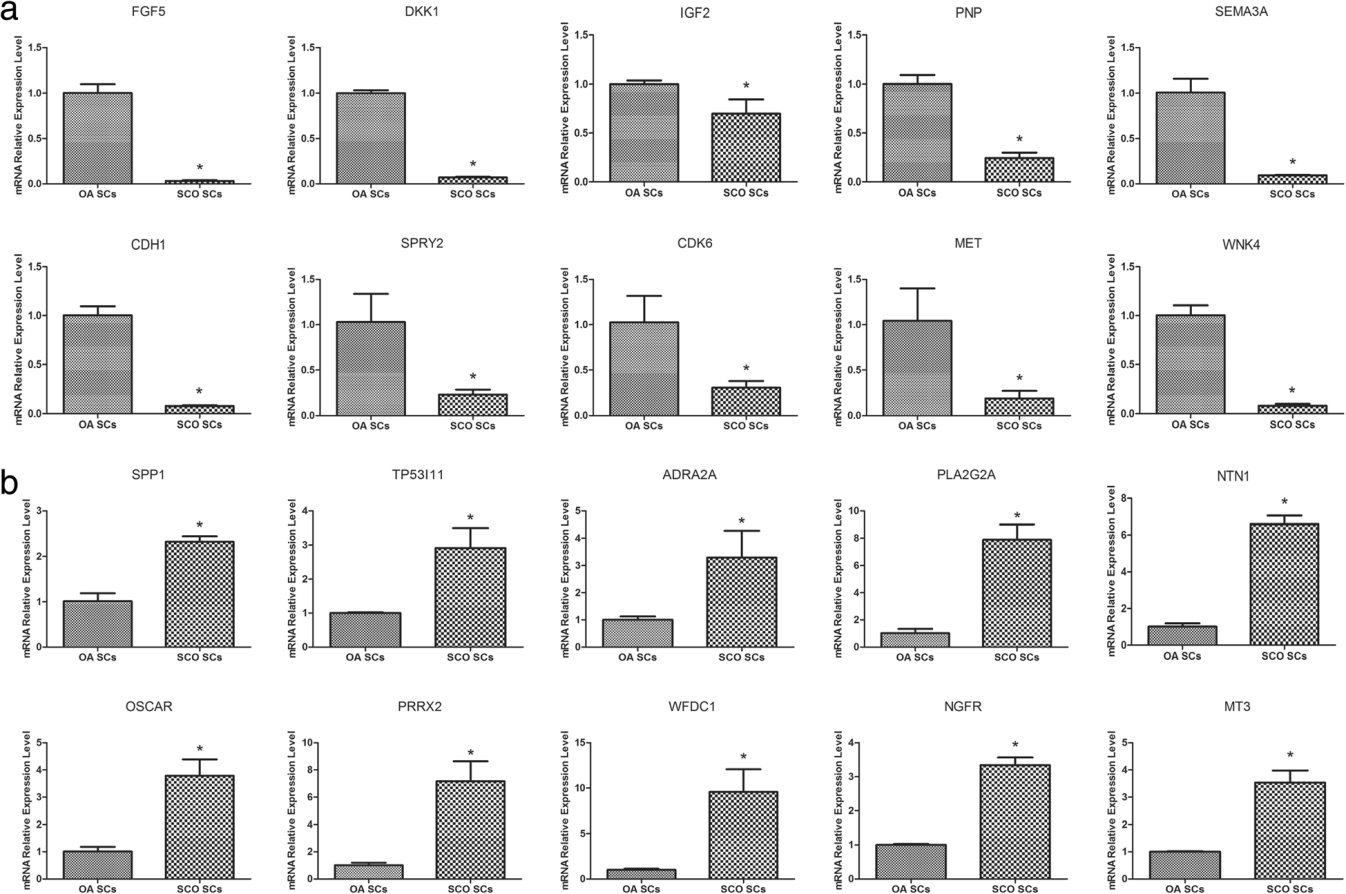
Differentially expressed genes in RNA-Seq were confirmed by Q-PCR. A total of 20 genes, including 10 SCOS Sertoli cells downregulated (*FGF5*, *DKK1*, *IGF2*, *PNP*, *SEMA3A*, *CDH1*, *SPRY2*, *CDK6*, *MET* and *WNK4*) (**a**) and upregulated (**b**) ones (*SPP1*, *TP53I11*, *ADRA2A*, *PLA2G2A*, *NTN1*, *OSCAR*, *PRRX2*, *WFDC1*, *NGFR* and *MT3*), from 308 DEGs were selected to validate the results of RNA-Seq. Note: OA obstructive azoospermia, SCOS Sertoli cell-only syndrome, SC Sertoli cell. Asterisk indicated statistically significant differences (*P* < 0.05)

In order to validate the RNA-Seq results, quantitative real-time PCR was performed to detect ten upregulated and ten downregulated expressed genes. Quantitative real-time PCR revealed that *BMP2*, *DKK1*, *FGF5*, *IGF2*, *MET*, *PNP*, *SEMA3A*, *SPRY2*, *WNK4*, and *TJP2* were statistically downregulated in human Sertoli cells of SCOS patients compared to OA patients (Fig. 3a). In contrast, *ADRA2A*, *CFD*, *MT3*, *NGFR*, *NTN1*, *OSCAR*, *PRRX2*, *SPP1*, *TP53I11*, and *WFDC1* were statistically upregulated in human Sertoli cells of SCOS patients compared to OA patients (Fig. 3b). The data of real-time PCR were fully consistent with the expression patterns of these *mRNAs* by RNA-Seq. These results revealed the credibility and validation of RNA-Seq results.

### Ontology analysis of differentially expressed genes

**Fig. 4:**
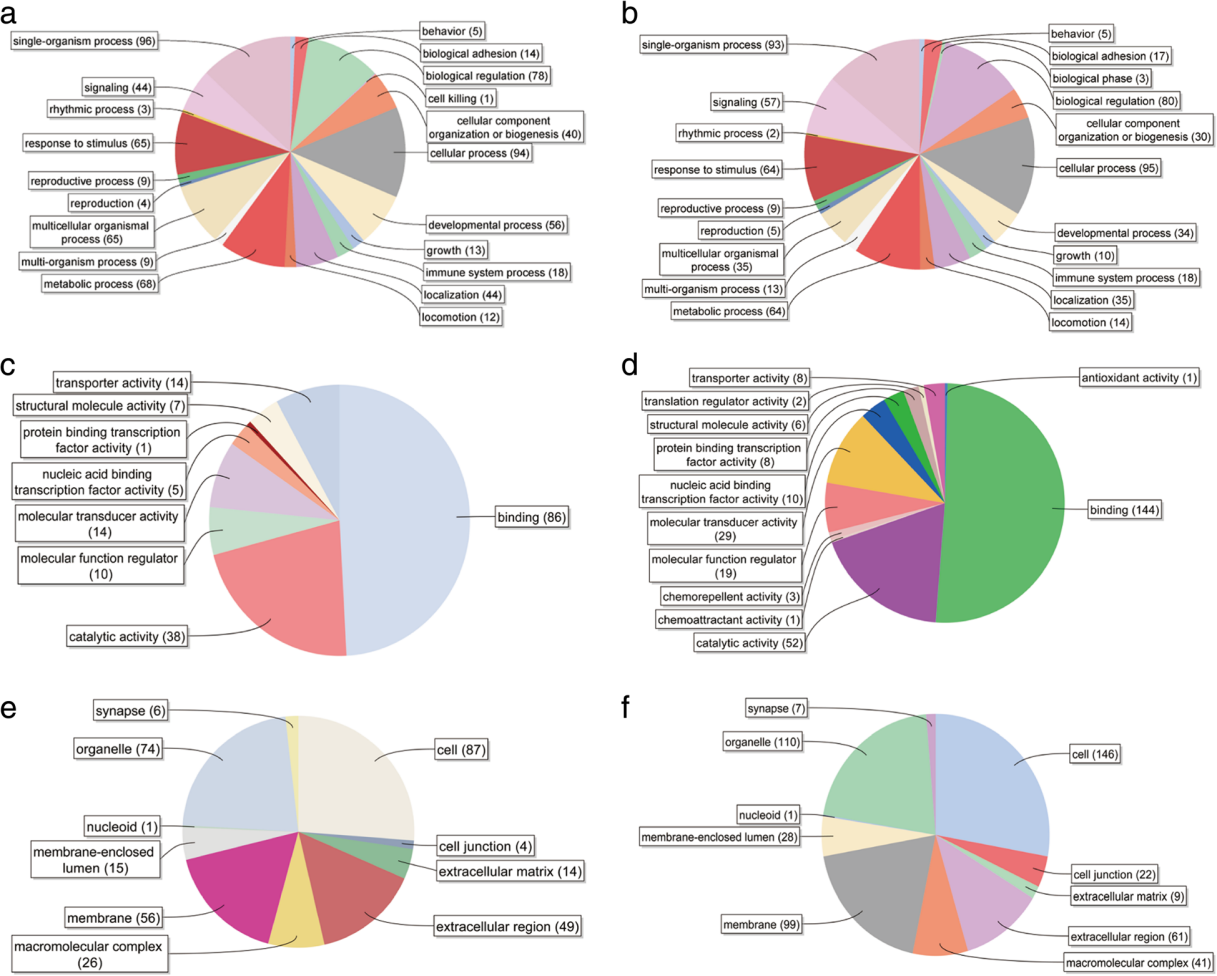
GO analysis of differentially expressed genes. **a**, **b** Biological process category of upregulated and downregulated genes in SCOS Sertoli cell. **c**, **d** Molecular function category of upregulated and downregulated genes in SCOS Sertoli cells. **e**, **f** Cellular component category of upregulated and downregulated genes in SCOS Sertoli cells

Blast2GO was employed for functional and gene ontology annotation on the differential expression genes (Fig. 4). As for the biological process analysis, 4 genes were annotated with reproduction, 9 were annotated with reproductive process in SCOS Sertoli cell upregulated genes, while 5 and 9 genes were linked with the reproduction and reproductive process in SCOS Sertoli cell downregulated genes, respectively.

As for the molecular function analysis, of 126 upregulated genes in SCOS Sertoli cells, 86 genes were annotated with binding, including protein binding (62), small molecule binding (15), extracellular matrix binding (2), lipid binding (8), and ion binding (38). Furthermore, 14 genes were involved with receptor activity, 1 conferred protein binding transcription factor activity. Of 182 downregulated genes in SCOS Sertoli cells, 141 genes were related with binding, including protein binding (119), small molecule binding (29), extracellular matrix binding (2), lipid binding (7), oxygen binding (2), and ion binding (62). Furthermore, 24 genes were annotated with receptor activity, and 9 were annotated with protein binding transcription factor activity.

As for cellular components, the amounts of SCOS Sertoli cells upregulated genes were annotated with cell (87), organelle (74), and membrane (56). There were 4 genes annotated with cell junction. As to the downregulated genes in SCOS Sertoli cells, there were 146, 110, and 99 genes annotated with cell, organelle, and membrane, respectively. We also noted that 21 genes were annotated with cell junction.

These genes, which were involved in transcription factor activity, binding, reproduction, etc., were postulated to be crucial for SSCs self-renewal and differentiation. The abnormal expressions of these genes may contribute to spermatogenesis disruption, eventually leading to SCOS.

### KEGG pathway analysis of differentially expressed genes

DAVID was applied to analyze DEG pathway, which was based on KEGG (Kyoto Encyclopedia of Genes and Genomes) pathway database. The detailed enriched pathway information was shown in Table 3. KEGG pathway analysis of these DEGs in human Sertoli cells showed abnormalities of several signaling pathway, including TGF-β signaling pathway and Hedgehog signaling pathway. There were 9 genes enriched in TGF-β signaling pathway, of which 4 were upregulated and 5 were downregulated in SCOS Sertoli cells. These results suggested that TGF-β signaling pathway could play key roles in human Sertoli cell regulations.

**Table 3:** Pathways involved in the RNA-Seq of human Sertoli cells from OA and SCOS testes

Term	Genes (%)	*P* value	Fold enrichment	FDR	Fisher exact
TGF-beta signaling pathway	9 (3.1)	4.1E−4	4.9	4.7E−1	7.5E−5
Hedgehog signaling pathway	5 (1.7)	2.9E−2	2.9	2.8E1	6.1E−3
Pathways in cancer	13 (4.5)	3.9E−2	4.5	3.7E1	1.9E−2
Bladder cancer	4 (3.2)	5.6E−2	1.4	4.8E1	1.1E−2
Oocyte meiosis	5 (2.1)	7.9E−2	2.6	6.1E1	2.8E−2
ECM-receptor interaction	6 (1.7)	9.7E−2	2.8	6.9E1	3.1E−2

### The expression of FGF5 is reduced in Sertoli cells from SCOS patients

**Fig. 5:**
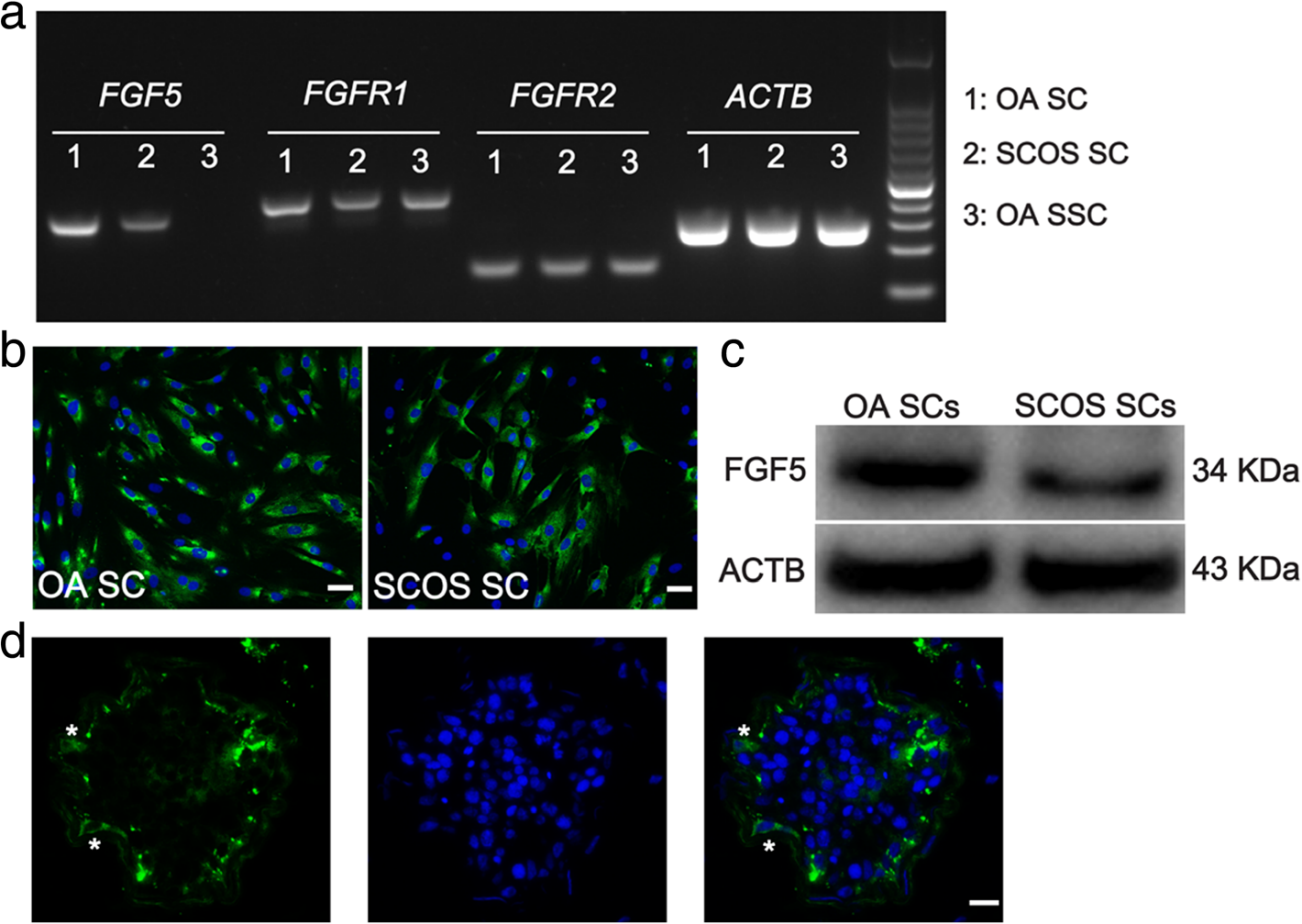
Expression of FGF5 in the human testis of OA and SCOS patients. **a** RT-PCR showed the expression of *FGF5*, *FGFR1*, and *FGFR2* in freshly isolated human Sertoli cells. ACTB was used as a loading control of total RNA. **b** Immunocytochemistry showed FGF5 expression in human Sertoli cells of OA and SCOS patients. **c** Western blot confirmed that expression of FGF5 was decreased at protein level in Sertoli cells from SCOS patients. **d** IHC revealed the location of FGF5 expression in the human testis (scale bar = 10 μm). Asterisk indicated the location of FGF5 expression in the human testicular section

As mentioned above, the expression of *FGF5* was verified to be decreased in Sertoli cells from SCOS patients. We further confirmed FGF5 protein and its receptor *FGFR1* and *FGFR2* expressions in human Sertoli cells (Fig. 5a, c). Immunocytochemistry demonstrated FGF5 expressed in Sertoli cells from both OA and SCOS patients (Fig. 5b). Western blot confirmed that expression of FGF5 was decreased at protein level in Sertoli cells from SCOS patients (Fig. 5c). Immunohistochemistry results showed that FGF5 was secreted by Sertoli cells in the human testis (Fig. 5d). These findings indicated that FGF5 may be a key factor secreted by human Sertoli cells and regulate the fate of SSCs via paracrine pathway.

### FGF5 promotes the proliferation of mouse SSCs

**Fig. 6:**
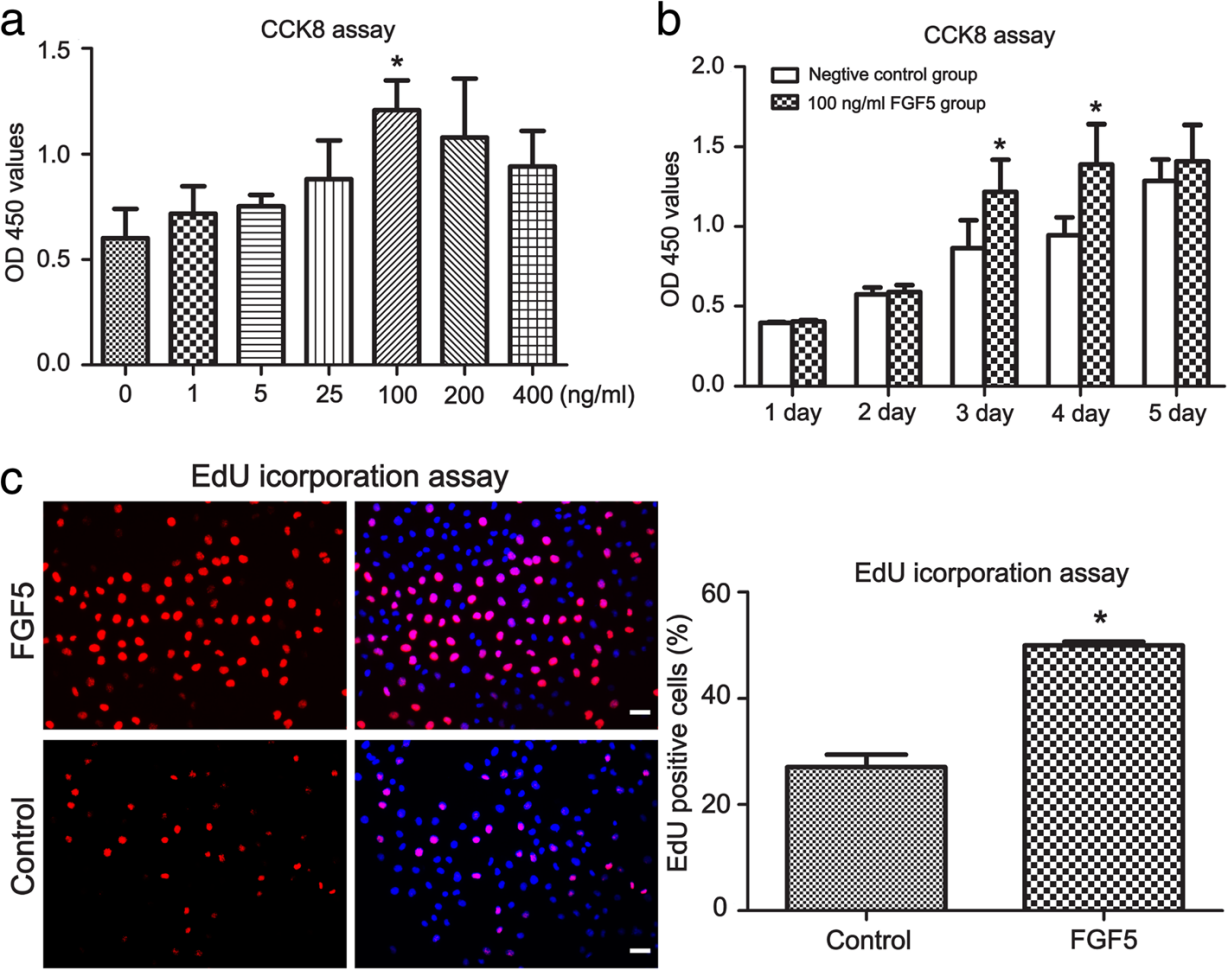
The effect of FGF5 in the proliferation of C18-4 (mouse SSCs line). **a** In CCK-8 assay, the growth of C18-4 cell line with various concentrations of FGF5 for 3 days (0, 1, 5, 25, 100, 200, and 400 ng/ml). **b** CCK-8 showed the growth activity of C18-4 cell line after treatment of FGF5 (100 ng/ml) for 1–5 days. **c** EdU incorporation assay revealed the proliferation activity after treatment of FGF5 (100 ng/ml) for 3 days. Asterisk indicated statistically significant differences (*P* < 0.05) between treatment of FGF5 and with the control

Since it is difficult to culture human SSCs in vitro, we further explored the function of FGF5 in the regulation of SSCs using mouse SSCs cell line C18-4. The cells were treated with varied concentrations of rFGF5 (0, 1, 5, 25, 100, 200, and 400 ng/ml) or different time periods (1, 2, 3, 4, and 5 days). CCK8 measurements showed that 100 ng/ml rFGF5 significantly increased cell numbers. We further explore rFGF5’s effect at the concentration of 100 ng/ml and found that rFGF5 significantly promotes cell proliferation when being added in the culture medium after 24 and 48 h (Fig. 6a, b). Furthermore, the results were confirmed by EdU incorporation assay. The percentage of EdU-positive cells in the rFGF5-treated group was significantly higher when compared with that in the control group (Fig. 6c). These results showed that rFGF5 promotes the proliferation of C18-4 in a dose- and time-dependent manner.

### The effects of FGF5 on phosphoprotein profiles in C18-4 cells

**Fig. 7:**
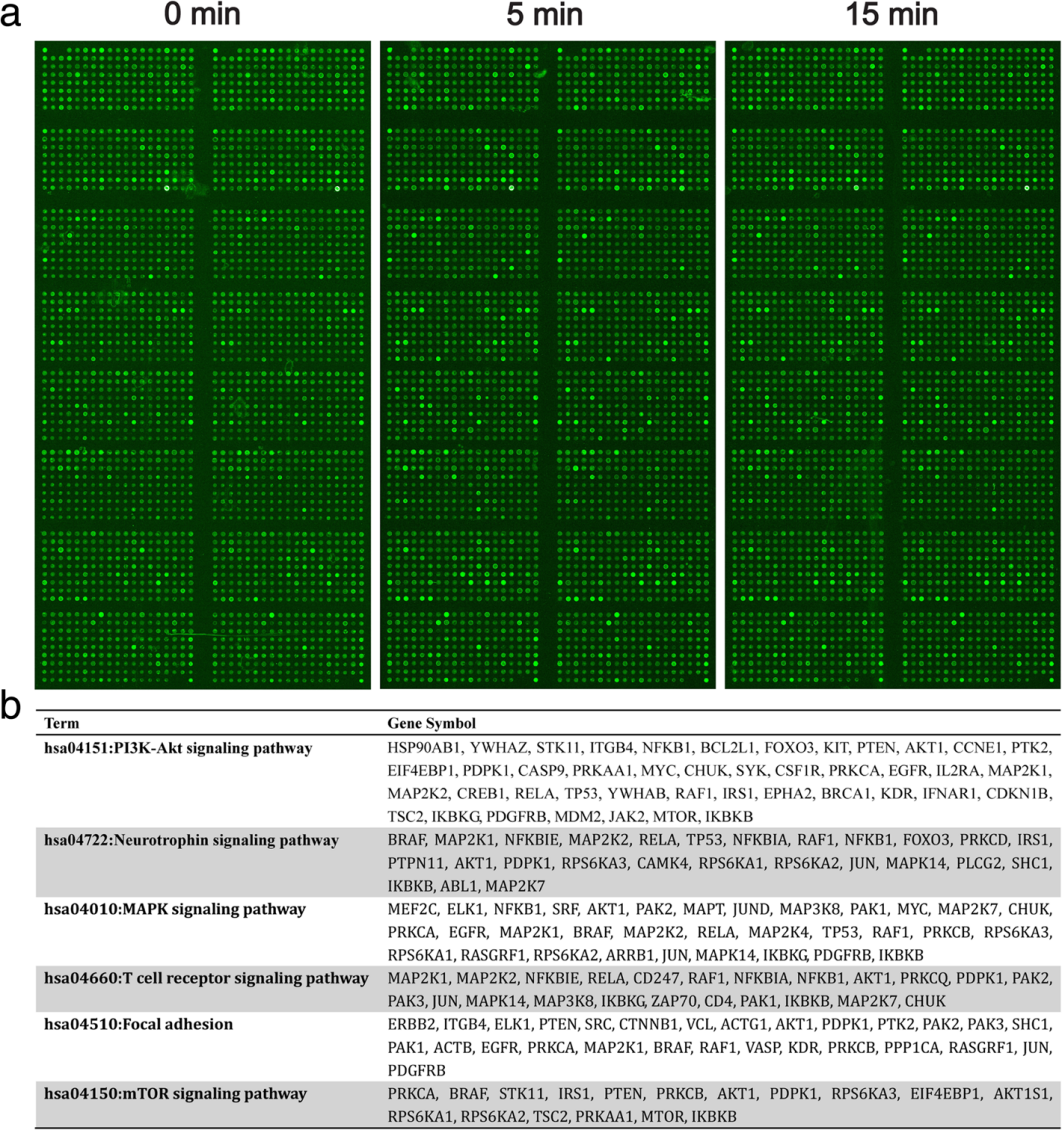
Phosphoprotein profiles in C18-4 cells after treatment of 100 ng/ml FGF5 in 5 and 15 min. **a** Distinct phosphoprotein profiles in C18-4 cells after 100 ng/ml FGF5 in 5- and 15-min treatment compared with the control. **b** KEGG informatics analysis showed the pathway enrichment of differentially phosphorylated proteins after treatment of 100 ng/ml FGF5 in 5 and 15 min compared with the control

The phospho antibody microarray was used to identify differences in the phosphoprotein profiles in C18-4 cells in response to rFGF5. Therefore, we identified differentially expressed phosphoproteins in rFGF5-treated C18-4 cells. We screened 1318 proteins on the phospho antibody microarray slides and obtained a total of 186 phosphoproteins that were significantly up- or downregulated more than 1.3-fold in response to 100 ng/ml rFGF5 after 15 min (*P* < 0.05) (Fig. 7a, b). Further analysis indicated the differentially expressed protein enrichment in different signaling pathways (Fig. 7a, b).

### FGF5 stimulated the proliferation of C18-4 cells via the activation of ERK and AKT

**Fig. 8:**
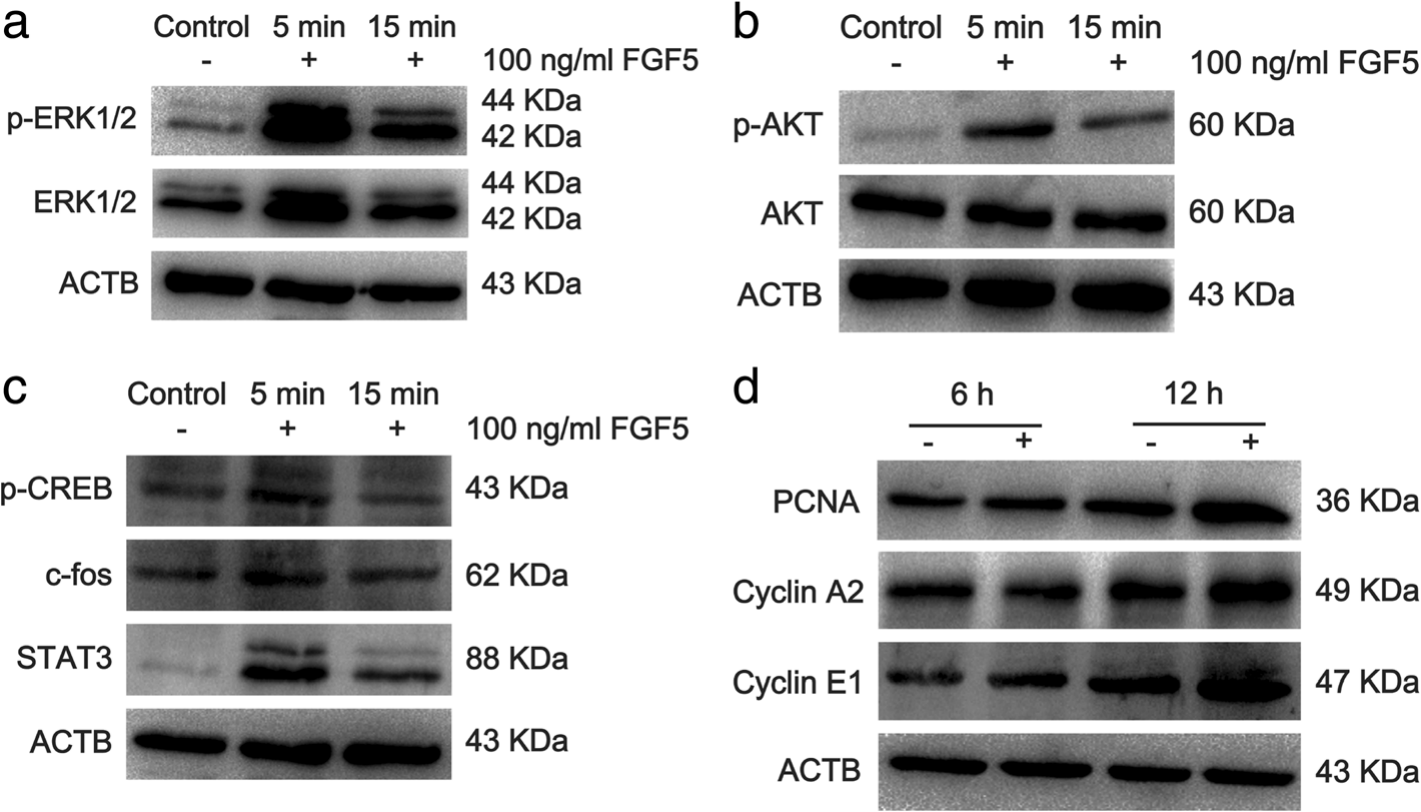
The expression changes of ERK, AKT, p-CREB, c-fos, STAT3, PCNA, Cyclin A2, and Cyclin E after treatment of FGF5. **a**, **b** Western blot revealed the expression changes of p-ERK and p-AKT after 100-ng/ml FGF5 treatment. ERK, AKT, and ACTB were used as loading controls. **c** The expression changes of p-CREB, c-fos, and STAT3 after treatment of FGF5 for 5 and 15 min. **d** The expression changes of PCNA, Cyclin A2, and Cyclin E after 100 ng/ml FGF5 treatment for 6 and 12 h

We noted that amounts of proteins were enriched in PI3K-AKT and MAPK signaling pathway (Fig. 7b), both of which were involved in lots of cellular events and biological processes. We further confirm that the expression profiles of p-ERK and p-AKT were dramatically increased in rFGF5-treated group than those in the control group after 5- and 15-min treatment. In addition, p-CREB, c-fos, and STAT3 were also remarkably elevated by rFGF5 (Fig. 8a–c). Since cell cycle proteins play crucial roles in regulating the entrance of cells to the S phase and cell proliferation, we further examined whether the expression levels of cell cycle regulators were changed during this process. Western blot revealed that the expression of PCNA, Cyclin A2, and Cyclin E1 was elevated after rFGF5 treatment (Fig. 8d). Collectively, these results indicated that FGF5 could activate ERK and AKT and stimulate PCNA, Cyclin A2, and Cyclin E1, leading to proliferation of SSCs.

## Discussion

**Fig. 9:**
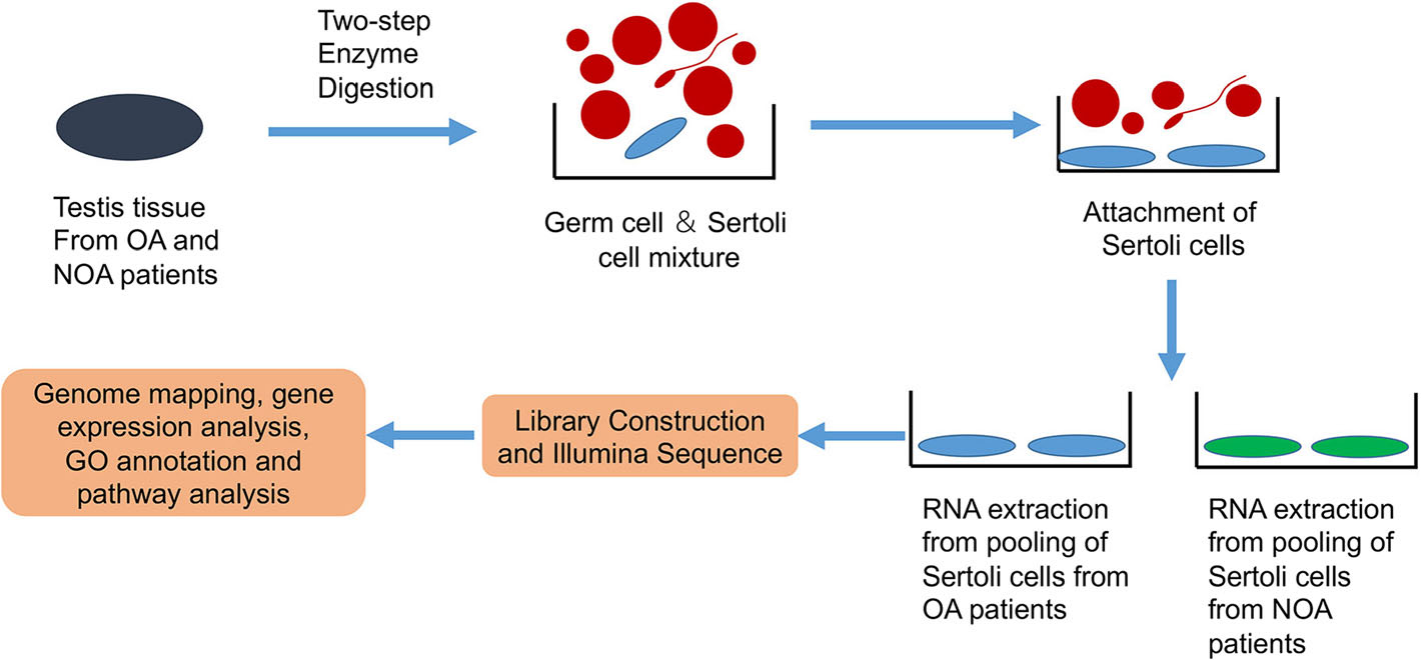
The schematic diagram shows sample processing of human Sertoli cells from OA patients and NOA patients, cell pooling, and RNA-Seq data processing procedure

Infertility is a worldwide reproductive health problem which affects 10–15% couples, about half of which are due to defects in male counterpart [1]. However, the pathological mechanisms, diagnosis, and treatment of male infertility have been still a challenge in clinic [27–30]. Large amounts of studies indicated that spermatogenesis is a complex process which involves Sertoli cell-germ cell interactions [9]. This process is strictly regulated at transcriptional levels. Nevertheless, only few genes or regulating factors have been found to be crucial for maintaining human Sertoli cell functions and spermatogenesis [3, 4, 9, 10]. Generating and comparing gene expression profiles in human Sertoli cells from normal and abnormal spermatogenesis testes is a prerequisite for complete understanding of their roles in the regulation of spermatogenesis. Although human testis transcriptome had been studied extensively using microarray assay or EST in the past several years, no systematic study has been reported on mRNA profiling in human Sertoli cells [16, 22, 31, 32]. In this study, we explored the gene expression profiles of human Sertoli cells from OA and SCOS patients using RNA-Seq techniques (Fig. 9). And we identified FGF5 was a crucial modulator of SSCs proliferation.

Using differential attachment method, we isolated human Sertoli cells from both OA and SCOS patients. Our previous report demonstrated that the isolated human Sertoli cells could be stably passaged for ten times without morphology and phenotype changes [33]. Despite these findings, we still chose the primary isolated Sertoli cells in our RNA-Seq study, to eliminate any unpredictable effects on the analysis. In this study, we detected the mRNA expressions of *GATA4*, *WT1*, *ABP*, and *FSHR*. Besides, we confirmed the expression of GATA4 using immunocytochemistry. All these results demonstrated that the freshly isolated cells were supposed to be human Sertoli cells with high purity.

In this study, we tried to explore DEGs between Sertoli cells from OA and SCOS patients, aiming to discover the potential modulator of spermatogenesis and underlie the molecular mechanisms of male infertility. In total, 308 DEGs were found between these two sources of Sertoli cells. We supposed that all these genes could play important roles in modulating Sertoli cell functions and spermatogenesis. Furthermore, we postulated that abnormal expression of these genes would lead to degenerated spermatogenesis and male infertility. In addition, these genes may be expected to be the biomarkers for the male infertility diagnosis. A confirmation of the RNA-Seq data is the necessary prerequisite to these scientific considerations [19, 34, 35]. Regarding this issue, Q-PCR was conducted to validate the DEGs, and the results showed that all the selected genes showed consistent pattern as RNA-Seq data. It indicated that the mRNA expression profiles in Sertoli cells from the testis of OA patients and SCOS patients obtained by RNA-Seq was reliable.

The GO term annotation indicates that the DEGs are associated with reproduction, biological adhesion, binding, developmental process, cell growth, etc. Furthermore, KEGG pathway analysis indicated that the DEGs were involved in the TGF-beta signaling pathway, Hedgehog signaling pathway, oocyte meiosis, ECM-receptor interaction, etc.

Among these DEGs, FGF5 was significantly downregulated in SCOS Sertoli cells. And this member of FGF family was deemed to only signal through FGFR1 and FGFR2. FGF5 was expressed in varied kinds of tissues and numerous species. And it played crucial roles in amounts of cellular events and biological process. In the reproductive system studies, FGF5 showed specific expression in Sertoli cells in mouse testes [36]. Another study demonstrated that FGF5 was expressed extremely higher in the testis compared with those in the liver and pancreas in Cashmere goat, indicating it may be involved in the regulation of spermatogenesis [25]. In our study, FGF5 was verified to be significantly expressed higher in Sertoli cells from males with normal spermatogensis, compared with SCOS patients. This finding suggested that it may contribute to the niche and regulate the fate of SSCs and spermatogenesis. However, the biological role of FGF5 in the male reproductive system has yet to be shown.

In the previous studies, it has been known that FGF5 acted as a mitogen to stimulate the proliferation of mesenchymal fibroblasts that contributed to the formation of connective tissues during embryonic development [23]. Another study showed that overexpression of FGF5 significantly promoted cell proliferation and colony formation of liver cell lines in vitro, while downregulation of FGF5 exhibited the opposite effect [37]. In the stem cell study fields, it has been demonstrated that supplementation with FGF5 in the culture medium stimulated the proliferation of equine umbilical cord blood stem cells (UCBSCs) in vitro [38]. A recent study showed that human tonsil-derived mesenchymal stem cells (T-MSCs) proliferated faster than adipose tissue-derived mesenchymal stem cells (A-MSCs) and bone marrow-derived mesenchymal stem cells (BM-MSCs) Interestingly, FGF5 displayed significantly elevated expression levels in T-MSCs compared with those in A-MSCs and BM-MSCs. In addition, siFGF5 transfection of T-MSCs led to a significant decrease in the proliferation rates, while administration of rFGF5 following siFGF5 treatment was found to reverse this effect [39]. It is demonstrated that GDNF and FGF2 were essential in in vitro culture of both mouse and human SSCs [40, 41]. And FGF2 could promote mouse SSCs self-renewal independent of GDNF supplement in vitro through MAP2K1 activation [42, 43]. Also, FGF9 could induce the mouse SSCs proliferation independently [44]. FGFR2 and FGFR3 were the common receptors of FGF2 and FGF9. However, compared with FGFR2 and FGFR3, FGFR1 deficiency showed more severe effect in the proliferation of mouse SSCs independent of GDNF supplement, suggesting that there may be other FGFs in the regulation of mouse SSCs proliferation [42]. Herein, FGF5 was significantly downregulated in SCOS Sertoli cells compared with OA Sertoli cells. And FGFR1 was the major receptor of FGF5 [45]. Therefore, we hypothesized that FGF5 plays an important role in the regulation of SSCs proliferation via similar pathway. In our study, we found that C18-4 showed an increased proliferation capacity after the exogenous administration of rFGF5. We speculated that FGF5 may regulate human SSCs proliferation and maintain SSCs pool and spermatogenesis. The reduced production of FGF5 in Sertoli cells may contribute to the exhaustion of SSCs pool and lead to SCOS.

FGFs control the proliferation and differentiation of several types of stem cells through the activation of several well-characterized intracellular signaling pathways, including MAPK and PI3K pathways. Previous studies have shown that FGF5 facilitates UCBSCs and T-MSCs proliferation through ERK1/2 activation [38, 39]. We deemed that the predominant signaling pathway activated by FGFs may depend on different sources of cells and on varied biological events. As mentioned above, rFGF5 promoted the proliferation of SSCs via PI3K-Akt signaling pathway and MAPK signaling pathway. PI3K-Akt pathway is an intracellular signaling pathway important in regulating the cell cycle. There are many known factors, including FGF family, IGF, and VEGF, that can activate PI3K-Akt pathway. PI3K-Akt pathway was essential to the proliferation of SSCs. Akt was phosphorylated after an addition of GDNF in mouse SSCs. Furthermore, conditional activation of Akt without the addition of GDNF also could induce the proliferation of SSCs, suggesting PI3K-Akt pathway was a prerequisite for the self-renewal of mouse SSCs. Moreover, MAPK signal pathway was important in the regulation of cell proliferation. Treatment of GDNF could induce the proliferation of mouse SSCs via MAPK signal pathway. However, addition of PD98059, a specific inhibitor of MEK1, could inhibit the cell proliferation after treatment of GDNF, indicating that MAPK signal pathway was essential in the self-renewal of mouse SSCs. Altogether, FGF5 could induce the propagation of mouse SSCs through activation of PI3K-Akt and MAPK signal pathways.

In conclusion, for the first time, we have analyzed the gene expression profiles in human Sertoli cells from the testis of OA and SCOS patients by high-throughput RNA-Seq technologies. Q-PCR analysis confirmed the expression profiles of these mRNAs in these two kinds of cells. The GO term and KEGG pathway annotations for these genes further illustrate the likely roles of these genes in Sertoli cells and spermatogenesis. Among these DEGs, FGF5 which was downregulated in SCOS Sertoli cells maybe a novel regulator in proliferation of SSCs. It was verified that FGF5 could induce the proliferation of mouse SSCs through PI3K-Akt pathway and MAPK pathway. Thus, this study shed novel insights in the regulation of the self-renewal and proliferation of SSCs and might offer new targets for the treatment of azoospermia.

## Abbreviations

ACTB: Actin beta
ART: Assisted reproductive technology
CASA: Computer-aided sperm analysis
DEGs: Differentially expressed genes
ESCs: Embryonic stem cells
FGF5: Fibroblast growth factor-5
FSH: Follicle-stimulating hormone
iPSCs: Induced pluripotent stem cells
LH: Luteinizing hormone
MA: Maturation arrest
micro-TESE: Microdissection testicular sperm extraction
NOA: Non-obstructive azoospermia
OA: Obstructive azoospermia
PFA: 4% paraformaldehyde
ROSI: Round spermatid injection
RS: Round spermatids
SCOS: Sertoli cell-only syndrome
SSCs: Spermatogonial stem cells
SSR: Surgical sperm retrieval
T: Testosterone
